# Digital health literacy and health behaviors of eighth and ninth graders from Germany

**DOI:** 10.1093/eurpub/ckac129.322

**Published:** 2022-10-25

**Authors:** K Dadaczynski, K Rathmann, J Schricker, L Bilz, G Sudeck, S Fischer, O Janiczek, E Quilling

**Affiliations:** 1 Public Health Center, Fulda University of Applied Sciences, Fulda, Germany; 2 Brandenburgischen Technischen Universität Cottbus, Cottbus, Germany; 3 Faculty of Economics and Social Sciences, University of Tübingen, Tübingen, Germany; 4 HAGE, Frankfurt, Germany; 5 Department of Applied Health Sciences, University of Applied Sciences, Bochum, Germany

## Abstract

**Background:**

Due to the high availability and use of digital media, health-related information is increasingly shifting into the digital space. While there are increasing empirical findings on general health literacy (HL), there is a lack of evidence on digital HL in adolescence and its association with health behavior.

**Methods:**

A cross-sectional study of 490 secondary school students (grades eight and nine) from the federal state of Hesse was conducted from October 2019 to February 2020. Digital HL was assessed using five subscales of the Digital Health Literacy Instrument (DHLI), while consumption of fruits, vegetables, soft drinks, and weekly physical activity were used as indicators of health behavior. In addition to gender and grade level, subjective social status (SSS) was used as a social characteristic. Univariate, bivariate, and multivariate analyses were performed, with binary-logistic regression adjusted for gender and SSS.

**Results:**

Across all items, the percentage of adolescents reporting difficulties in acquiring and dealing with digital health information ranges from 15.3 % to 37.5 %. Stratified by social characteristics, gender and socioeconomic differences were found with girls and respondents reporting a lower SSS more often showing a limited digital HL. Adolescents with moderate and low digital HL report higher levels of low physical activity, non-daily fruit and daily soft drink consumption. Depending on the health behavior, different relationship patterns can be observed for the dimensions of digital HL.

**Conclusions:**

The findings suggest a need for interventions to promote digital HL among adolescents, particularly for those of low SSS. In this context, the differential relationship patterns with health behaviors provide an avenue for the development of specific interventions.

